# Case Report: Atypical Manifestations Associated With FOXP3 Mutations. The “Fil Rouge” of Treg Between IPEX Features and Other Clinical Entities?

**DOI:** 10.3389/fimmu.2022.854749

**Published:** 2022-04-11

**Authors:** Micaela Gentile, Maurizio Miano, Paola Terranova, Stefano Giardino, Maura Faraci, Filomena Pierri, Enrico Drago, Daniela Verzola, Gianmarco Ghiggeri, Enrico Verrina, Andrea Angeletti, Barbara Cafferata, Alice Grossi, Isabella Ceccherini, Gianluca Caridi, Francesca Lugani, Lorenzo Nescis, Enrico Fiaccadori, Luca Lanino, Daniela Fenoglio, Edoardo La Porta

**Affiliations:** ^1^ Unitá Operativa (UO) of Nephrology, Dialysis and Transplantation, IRCCS Istituto Giannina Gaslini, Genoa, Italy; ^2^ Dipartimento di Medicina e Chirurgia, Università di Parma, Parma, Italy; ^3^ Hematology Unit, IRCCS Istituto Giannina Gaslini, Genoa, Italy; ^4^ Hematopoietic Stem Cell Unit, IRCCS Istituto Giannina Gaslini, Genoa, Italy; ^5^ Department of Internal Medicine and Medical Specialties, University of Genova, Genoa, Italy; ^6^ Dialysis Unit, Department of Pediatric, IRCCS Istituto Giannina Gaslini, Genoa, Italy; ^7^ Pathology Unit, IRCCS Istituto Giannina Gaslini, Genoa, Italy; ^8^ Unitá Operativa Semplice Dipartimentale (UOSD) Laboratory of Genetics and Genomics of Rare Diseases, IRCCS Istituto Giannina Gaslini, Genoa, Italy; ^9^ Laboratory on Molecular Nephrology, Division of Nephrology, Dialysis, and Transplantation, IRCCS Istituto Giannina Gaslini, Genoa, Italy; ^10^ Unitá Operativa (UO) of Nephrology, Dialysis, and Transplantation, Istituto di Ricovero e Cura a Carattere Scientifico (IRCCS) Ospedale San Martino, Genoa, Italy; ^11^ Unitá Operativa (UO) Nefrologia, Azienda Ospedaliera-Universitaria, Parma, Italy; ^12^ Department of Oncology and Hematology, Humanitas Clinical and Research Center, Milan, Italy; ^13^ Biotherapy Unit, Istituto di Ricovero e Cura a Carattere Scientifico (IRCCS) Ospedale San Martino, Genoa, Italy; ^14^ Centre of Excellence for Biomedical Research and Department of Internal Medicine, University of Genoa, Genoa, Italy; ^15^ Department of Internal Medicine, University of Genoa, Genoa, Italy

**Keywords:** FOXP3, ALPS, IPEX, membranous glomerulopathy, regulatory T cells, NGS

## Abstract

**Introduction:**

The Forkhead box protein P3 (FOXP3) is a transcription factor central to the function of regulatory T cells (Treg). Mutations in the *FOXP3* gene lead to a systemic disease called immune dysregulation, polyendocrinopathy, and enteropathy, an X-linked syndrome (IPEX) characterized by the triad of early-onset intractable diarrhea, type 1 diabetes, and eczema. An atypical presentation of IPEX has been reported.

**Method:**

We report rare cases with equivocal clinical associations that included inflammatory, kidney, and hematologic involvements screened with massively parallel sequencing techniques.

**Results:**

Two patients with hemizygous mutations of *FOXP3* [c.779T>A (p.L260Q)] and [c.1087A>G (p.I363V)] presented clinical manifestations not included in typical cases of IPEX: one was a 16-year-old male patient with an initial clinical diagnosis of autoimmune lymphoproliferative syndrome (ALPS) and who developed proteinuria and decreased kidney function due to membranous nephropathy, an autoimmune renal condition characterized by glomerular sub-epithelial antibodies. The second patient was a 2-year-old child with bone marrow failure who developed the same glomerular lesions of membranous nephropathy and received a bone marrow transplantation. High levels of IgG4 in serum, bone marrow, and kidney led to the definition of IgG4-related kidney disease (IgG4 RKD) in this young boy. The circulating Treg levels were normal in the former case and very low in the second.

**Conclusion:**

Two atypical associations of functional mutations of *FOXP3* that include ALPS and IgG4 RKD are described. Membranous nephropathy leading to renal failure completed in both cases the clinical phenotypes that should be included in the clinical panorama of *FOXP3* failure.

## Introduction

Immunologic disorders of genetic origin represent a group of diseases characterized by a wide spectrum of phenotypes that frequently pose a diagnostic challenge for possible clinical overlaps (1). They are, in general, monogenic disorders characterized by pleiotropic clinical manifestations, ranging from increased susceptibility to infections to significant immune dysregulation or autoimmunity and hematologic abnormalities, including lymphoproliferation and cytopenia (2). With the advent of next-generation sequencing (NGS), we were able to better classify these disorders based on the underlying mutations.

The immune dysregulation, polyendocrinopathy, and enteropathy X-linked syndrome (IPEX) is an inherited condition associated with the mutation of Forkhead box Protein P3 (FOXP3), a transcriptional factor uniquely expressed by CD4^+^CD25^+^ regulatory T cells (Treg) and closely implicated in the regulation of immune homeostasis (3, 4). IPEX is a life-threatening condition usually emerging in early childhood and characterized by the triad of early-onset intractable diarrhea, type 1 diabetes (T1D), and eczema (5). The spectrum of *FOXP3* mutations may, however, extend beyond the classical IPEX triad, with a still undefined number of cases with an atypical presentation, including late-onset involvement, mild disease phenotypes, and predominant hematologic clinical features (6).

Here we describe two cases with proven pathogenetic variants of *FOXP3* who presented the atypical signs reported in IPEX: a 16-year-old boy who developed an autoimmune lymphoproliferative syndrome (ALPS) with late onset and a 2-year-old child who presented an IgG4-related disease (IgG4 RD) (7) as the first symptom. Kidney involvement occurred at the second stage of the disease in both patients, with similar glomerular lesions of membranous glomerulopathy. The atypical clinical presentation associated with *FOXP3* mutations represents a new syndromic template that should be considered in clinical medicine.

## Patient 1

The patient is a 16-year-old male with a clinical diagnosis of ALPS (8). At the age of 8 years, he was diagnosed with chronic (> 6 months) lymphoadenopathy. A malignant disorder was excluded, and immunological screening showed high levels of TCR-αβ+-double-negative T-cells (DNTs) and a rise in vitamin B12 serum levels. According to the diagnostic criteria (8), he was diagnosed with ALPS, and the treatment with mycophenolate mofetil (MMF) at 1 g/m^2^/day was started with a partial response. At that time, the laboratory tests showed normal renal function and the absence of proteins in the urine.

However, in the following years, the disease symptoms were not completely controlled: at the age of 11, he developed severe thrombocytopenia (PLT 27,000/mm^3^) which was refractory to MMF therapy but successfully treated with sirolimus at 2 mg/m^2^/day.

**Figure 1:**
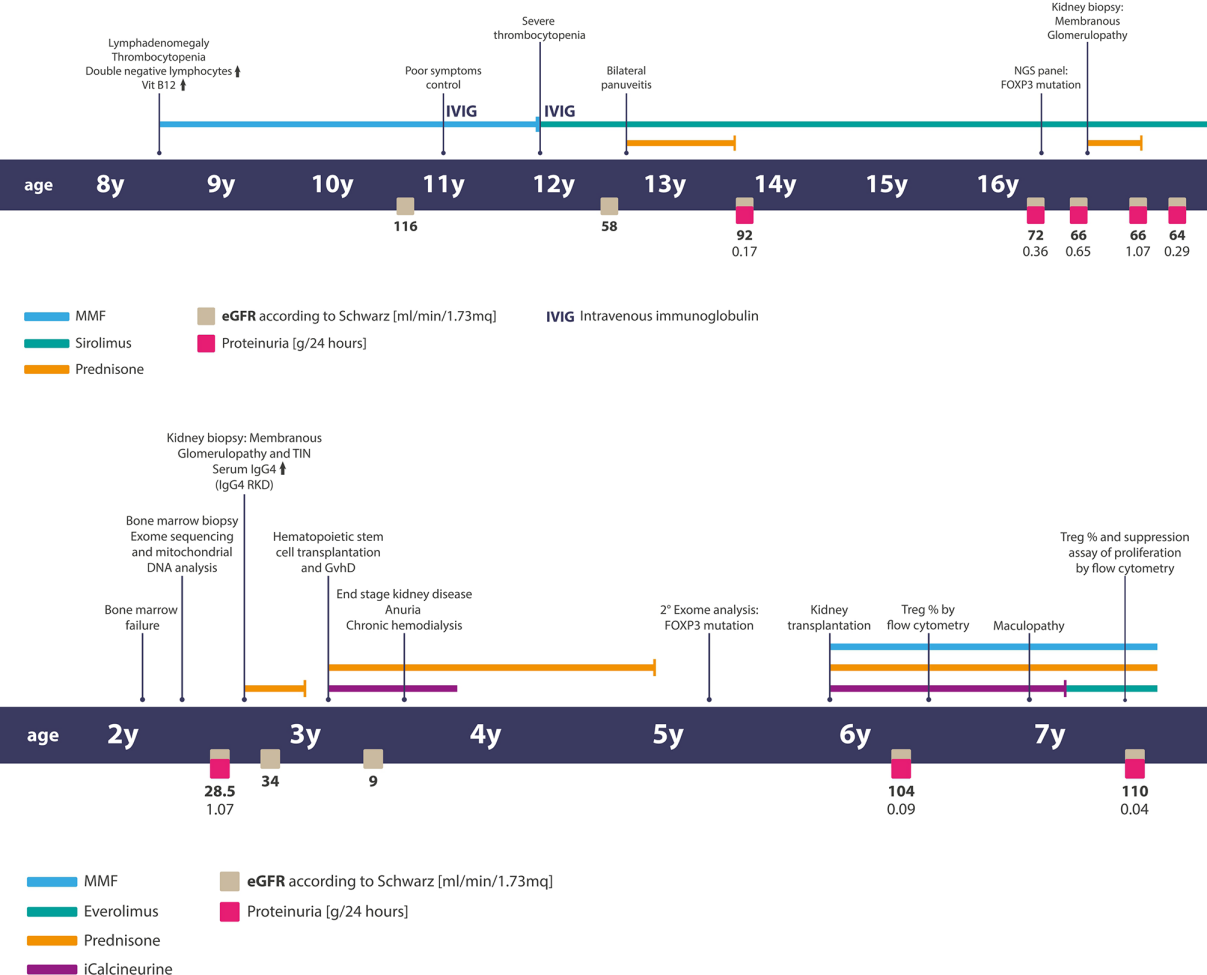
Timelines of clinical events, diagnostic exams, and treatments of patients. Above—the timeline of patient 1 and below— the timeline of patient two.

**Figure 2:**
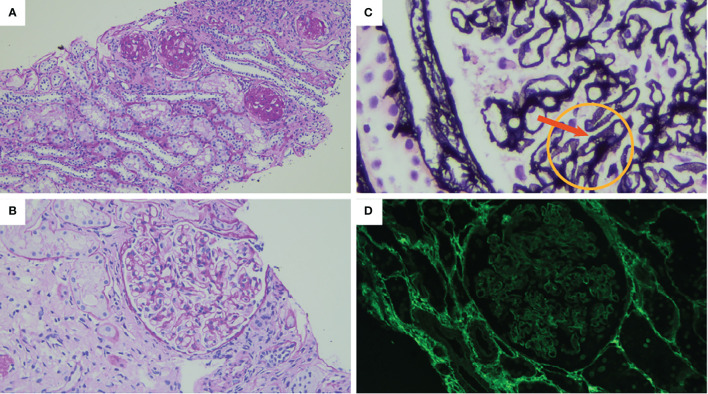
The result of the renal biopsy shows **(A)** diffuse global glomerulosclerosis. Periodic acid Schiff (PAS) stain original magnification: ×100. **(B)** Glomerular basal membrane (GBM) thickening. PAS stain original magnification: ×400. **(C)** Prominent vacuolated appearance of GBM indicated by the arrow. Jones methenamine silver, original magnification: ×1,000. **(D)** No PLA2R immune deposits (immunofluorescence, original magnification: ×400).

At the age of 12, he presented bilateral pan-uveitis treated with local therapy and prednisone, starting with 1 mg/kg/day and tapering until suspension at 1 year later. At the same time, the laboratory tests showed increased creatinine without any changes in the urine tests. The kidney function temporarily improved during steroid therapy but worsened later. Afterwards, he showed complete remission of ALPS-like clinical manifestations. At the age of 16, due to the appearance of proteinuria and persistence of decreased kidney function, the patient was admitted to the Nephrology Unit. A timeline of the events and treatments is presented in **Figure 1**. The serum testing was negative for antinuclear and anti-neutrophil cytoplasmic antibodies, and the serum complement was normal. A renal biopsy was performed. Light microscopy findings included a membranous pattern with 63% (7 out of 11) obsolete glomerulus (**Figures 2A–C**). Active lymphocytic inflammation was present in tubule–interstitium, especially near arterioles. Immunofluorescence microscopy showed glomerular deposition of IgG and C3. The M-type phospholipase A2 receptor (PLA2R) antibody was absent in serum, and the PLA2R antigen was absent in tissue as well as thrombospondin type 1 domain-containing 7A (THSD7A) antigen in tissue (**Figure 2D**). The IgG–IgG4 immunohistochemical staining on kidney biopsy was negative. All investigations are summarized in **Table 1**.

**Table 1:** Results of the investigations and comparison between the patients.

		Patient 1	Patient 2
Hematological and immunological investigations			
		■ Severe thrombocytopenia: 27 × 10^9^ L (reference values: 200–450) ■ Latero-cervical and abdominal lymphadenomegaly ■ Vitamine B12: 1,086 pg/ml (reference values: 191–663) ■ DNTs 2.7% (reference values <1.5)	■ Trilinear insufficiency requiring weekly transfusion of red blood cells and platelets ■ Serum IgG4: 353 mg/dl (reference values: <120) ■ BMB: severe hypocellularity (20%); lymphoplasmacytic infiltrate IgG4+ cells/hpf: 0–9 IgG+/IgG4+ ratio: 50% ■ Treg CD4+CD25+FOXP3+
**Phenotypic characterization of CD4+FoxP3+CD25 high**-**Treg lymphocytes** (reference values: 1–5%)		2.3%	0.3%
**Suppression activity by CD4+CD25 high**-**Treg lymphocytes** (reference values: >25%)		–	17%
**Nephrological investigations**			
**Kidney biopsy**–l**ight microscopy** (H&E, PASM, PAS, and Masson’s trichrome stains)		Membranous pattern with 7 out of 11 obsolete glomerulus. Active lymphocytic inflammation in tubule–interstitium, especially near arterioles	50% cortex and 50% medulla. Up to 40 glomerulus. Tubulointerstitial inflammatory infiltrate. Irregular thickening of the glomerular basement membranes and subepithelial deposits
**Kidney biopsy**–i**mmunofluorescence/IHC**		Glomerular deposition of IgG and C3. Anti-PLA2R, IgG/IgG4, and THSD7A negative	IgA, IgM, C4 and C1q: negative. IgG and C3 glomerular membrane and tubular deposits IgG deposits. IgG4+ cells/hpf: >10 IgG+/IgG4+ ratio: 100%
**eGFR** at the time of kidney biopsy (normal values: >90 ml/min)		66 ml/min/1.73 m^2^	28.5 ml/min/1.73 m^2^
**Proteinuria** at the time of kidney biopsy (normal values: <0.15 g/24 h)		0.65 g/24 h	1.07 g/24 h
**Next-generation sequencing**			
**FOXP3 mutation**	Leucine-Zipper Domain (exon 8) (NM_014009.3) [c.779T>A (p.L260Q)]		Fork-head domain (exon 11) (NM_014009.3) [c.1087A>G (p.I363V)]

BMB, bone marrow biopsy; DNT, double-negative lymphocytes; H&E, hematoxylin and eosin; IHC, immunohistochemistry; PAS, periodic acid-Schiff; PASM, periodic Schiff-methenamine silver.

In the light of histological diagnosis, we decided to re-validate the genetic evaluation of the patient that had been tested through an NGS-based gene panel, implementing the human phenotype ontology (HPO) code. We found a mutation at the leucine–zipper domain (exon 8) of the *FOXP3* gene (NM_014009.3): c.779T>A (p.L260Q), never previously reported on GnomAD database (https://gnomad.broadinstitute.org/) and predicted to be likely pathogenic by the Varsome (https://varsome.com/” https://varsome.com/) suite of variant annotation. The proband is hemizygous for the variant inherited from the asymptomatic mother. The patient does not present the classical triad of IPEX (gastrointestinal involvement, cutaneous manifestation, and polyendocrinopathy), and the number of Treg CD4+CD25+FOXP3+ was in the normal range (2.4%).

He was successfully treated with steroid that started at 1 mg/kg for 1 month with slow tapering and currently continues immunosuppressive therapy with sirolimus, with a good response on proteinuria and with stable kidney function.

## Patient 2

A previously healthy 2-year-old child was diagnosed with trilinear cytopenia due to bone marrow failure requiring weekly platelet and red blood cell transfusions. The bone marrow biopsy (BMB) showed a severe hypocellularity (20%) with lymphoplasmacytic infiltrate. The screening investigations performed to rule out the diagnosis of Fanconi anemia and telomeropathies were negative (DEB test, telomere length measurement). Congenital bone marrow failure syndromes (cBMFs) were also excluded by a large NGS panel, and the mitochondrial DNA analysis results were normal as well. The IgG subclass analysis showed elevated serum levels of IgG4 subclass. The timeline of the events and treatments is presented in **Figure 1**.

During hospitalization, kidney failure with tubular acidosis was also found. The urine analysis showed microhematuria, proteinuria (1 g/L), and granular casts. The renal ultrasound demonstrated no abnormal findings.

**Figure 3:**
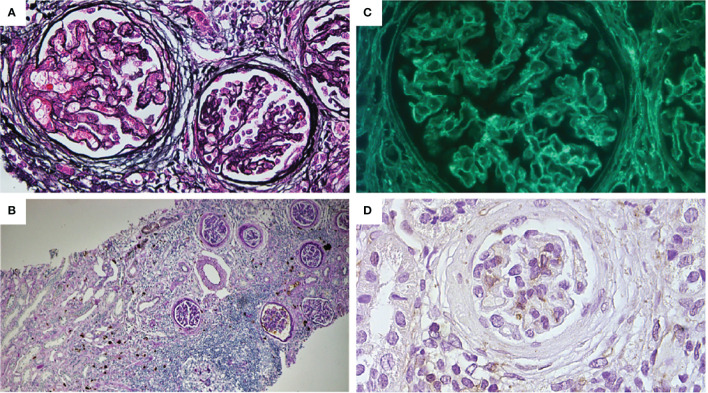
**(A)** Mild thickening of the capillary wall and glomerular basement membrane. Bowman capsule enlargement and reduplication (periodic Schiff-methenamine silver, original magnification: ×400). **(B)** Interstitial and peri-tubular IgG4 deposits. Mild tubular atrophy and interstitial fibrosis. Moderate focal and periglomerular lymphoplasmacytic infiltrate (PAS + IgG4 IHC, original magnification: ×100). **(C)** Glomerular sub-epithelial deposits with granular pattern (immunofluorescence for anti-PLA2R antibodies, original magnification: ×400). **(D)** Absence for THSD7A glomerular expression (IHC original magnification: ×200).

A renal biopsy was performed, and a kidney sample with up to 40 glomeruli was obtained (**Table 1**). The light microscopy showed tubulointerstitial inflammatory infiltrates (mainly composed of lymphoplasmacytic cells), irregular thickening of the glomerular basement membranes, and subepithelial deposits (**Figures 3A, B**).

Immunofluorescence showed subepithelial glomerular membrane IgG deposits with granular pattern and tubular wall deposits, C3 glomerular deposits, and focal tubular deposits.

A diagnosis of membranous glomerulopathy associated with tubule–interstitial nephritis was made. Immunohistochemical staining for IgG4 demonstrated plasma cells with a complete overlapping positivity for IgG and IgG4 (**Figure 3B**).

IgG–IgG4 Immunohistochemical staining was also performed on BMB, which showed a 50% overlapping positivity for IgG and IgG4. This finding, together with high circulating serum levels of IgG4, led to the definition of an IgG4-related kidney disease (IgG4 RKD).

After the diagnosis of IgG4 RKD, corticosteroid therapy (1 mg/kg/day), targeting both the hematological disorder and glomerulopathy, was started, without clinical response. Thereafter, at 8 months from the onset of the disease, the patient underwent hematopoietic stem cell *transplantation* (HSCT) from his HLA-identical 5-year-old healthy brother. The HSCT characteristics are shown in **Table 2**. During the early period after the infusion of bone marrow cells, the child developed severe gastro-intestinal and cutaneous toxic complications. Engraftment of neutrophils occurred 11 days after transplant, and full donor chimerism (100% donor cells) was demonstrated after the engraftment and confirmed over time. Furthermore, we observed many endothelial complications represented by two episodes of a severe veno-occlusive disease requiring treatment with defibrotide and paracentesis, thrombotic microangiopathy managed with discontinuation of CSA, and cycles of plasmapheresis and eculizumab. The patient developed acute GvHD (a-GvHD) on day +35, with a maximum of grade IV involving the skin, liver, and gastro-intestinal tract with hemorrhagic diarrhea as confirmed by intestinal biopsies. This condition was refractory to steroids at 2 mg/kg, and its management required a high dose of methylprednisolone and etanercept and ileostomy placement for intestinal sub-occlusion. Etanercept and prolonged steroid therapy enable the complete remission of a-GvHD. The evaluation of immunological reconstitution at 1 year after HSCT demonstrated a value of lymphocyte subpopulations within the normal range. After HSCT, the patient experienced severe renal failure triggered by the renal toxic effect of antiviral and antibiotic therapies by septic shocks and by endothelial damage related to transplant. The decrease of renal function required kidney replacement therapy through chronic hemodialysis.

**Table 2:** Hematopoietic stem cell transplantation (HSCT) features and related main complications of patient 2.

Transplant’s features	
**Main indication to HSCT**	Bone marrow failure (failure of first-line treatment with Eltrombopag and GCSF)
**Age at HSCT**	3 years and 9 months
**Stem cell donor**	Matched related donor (brother)
**Stem cell source**–**cell dose**	Bone marrow - MC 10.7 × 10^8^ per kilogram of recipient’s weight
**Conditioning regimen** **(cumulative dose)**	­- Treosulfan 42 gr/m^2^ ­- Fludarabine 160 mg/m^2^ ­- Thiotepa 8 mg/kg ­- ATG 10 mg/kg
**GvHD prophylaxis**	­- CyA ­- MMF

ATG, antithymocyte globulin; CyA, cyclosporin A; GCSF, granulocyte colony-stimulating factor; HSCT, hematopoietic stem cell transplantation; MC, mononuclear cells; MMF, mycophenolate mofetil; MPD, methylprednisolone.

“In consideration of the rarity of the incidence of IgG4 RD in children, we speculated that it could be an epiphenomenon of a hereditary disease. After HSCT, the patient experienced severe clinical issues related to immunosuppression and GvhD that required intensive care and led to a further decrease of renal function, with the need for kidney replacement therapy through chronic hemodialysis. Moreover, in the following years, he presented several complications, such as alopecia, candida infection with hepatic involvement, hyper eosinophilia, hypoparathyroidism, and others.

In consideration of the above-mentioned clinical manifestations, only partially ascribable to both CKD and GvhD, a further genetic analysis was done. We performed whole-exome sequencing on the proband, using a pre-transplant blood sample, and his relatives (both parents and the asymptomatic brother), using HPO codes addressing also membranous nephropathy and IgG4 RKD. A hemizygous mutation in the Fork-head domain (exon 11) of the *FOXP3* gene (NM_014009.3), c.1087A>G (p.I363V), was found. This variant, inherited from the mother, had not been reported before in the GnomAD database. Nonetheless, it was predicted to be likely pathogenic by the Varsome website and was already described in literature (9); thus, a diagnosis of IPEX was made. Unexpectedly, the same variant was found in hemyzigosis also in the proband’s healthy brother, who had been his bone marrow donor. Therefore, we performed a flow cytometry analysis to evaluate the assessment of Treg (CD3+CD4+CD25+Foxp3+) that resulted normal for brother (2.7%) and mother (2.1%) but not in our patient (0.3%). Differently from the exome, a cytometry analysis was performed on post-bone marrow transplant blood sample.

To better characterize the pathogenesis and the kidney involvement of the *FOXP3* mutation in our clinical presentation, we performed indirect immunofluorescence in serum and immunohistochemistry (IHC) on kidney tissue for M-type PLA2R, that resulted highly positive, and THSD7A, that otherwise resulted negative (**Figures 3C, D**).

At the age of 5, the patient underwent deceased donor kidney transplant, and after more than 1 year, he presents normal kidney function and absence of proteinuria. At the age of 7, he was diagnosed with severe maculopathy secondary to multifactorial causes (previous CMV infection, microangiopathy damage, *etc.*), including calcineurine inhibitors. Thus, a therapeutic shift from tacrolimus to everolimus (started with 2 mg/m^2^/day) was made. Thereafter, we investigated again the Treg assessment, and we performed T cell proliferation suppression. The percentage of Treg was still low, and Treg suppression activity was significantly lower in our patient compared to that of his sibling (10) (**Supplementary Materials 1–3**).

## Discussion

Here we describe two children with *FOXP3* mutations and clinical manifestations mainly represented by variable hematological involvement at the onset, *i*.*e*., ALPS in one case and IgG4-related disease in the other, and similar kidney involvement occurring at the second stage of the disease that was membranous glomerulopathy in both cases. Overall, the clinical features associated with *FOXP3* mutations in the two children herein described did not fit a classical diagnosis of IPEX that is usually represented by the classic triad of intractable diarrhea, T1D, and eczema (5) with early-onset, usually under 3-5 years. The two clinical associations here described are therefore anecdotal for the wide spectrum of possible phenotypes associated with *FOXP3* mutations (11).

The first case presented a syndrome with late onset (16 years) in which an ALPS like hematologic condition predominated at the start. The second child presented severe cytopenia due to bone marrow failure at presentation and required weekly platelet and red blood cells transfusions. Molecular and cellular investigations (DEB test, telomere length measurement, etc.) did not allow for a more precise setting in any cBMFs and only highlighted the presence of high IgG4 levels in kidney, bone marrow and in serum. The renal syndrome predominated at a second stage, leading in both cases to the development of chronic renal failure and a similar pathologic involvement with membranous deposits of antibodies (PLA2R positive in only one case). Kidney biopsy features of the two cases are presented and compared in **Table 1**.

ALPS is a rare disorder with immune dysregulation characterized by early-onset, chronic, non-malignant lymphoproliferation, splenomegaly, and autoimmune manifestations due to defective lymphocyte apoptosis and elevations in CD3+TCRαβ+CD4−CD8− DNTs. Although the diagnosis of ALPS is based on clinical criteria and does not require the presence of any molecular defect (8), ALPS is established to be usually associated with mutations in genes involved in the apoptosis pathway (FAS, FASLG, and CASP10). Moreover, there is growing interest in considering a wide variability of ALPS-like disorders characterized by an expanded number of disease-associated genes (CASP8, NRAS, KRAS, CTLA4, LRBA, FADD, PRKCD, STAT1, STAT3, TNFRSF13B, ADA2, *etc.*) whose mutations result in some overlapping symptoms, including lympho-proliferation, cytopenia, inflammatory bowel disease, malignancy predisposition, and other autoimmune manifestations (12). *FOXP3* could now be added to the list above. Kidney involvement in ALPS is uncommon and rarely described (13–16), with no univocal spectrum of clinical features or histological lesions.

IgG4 RD is a recently recognized systemic immune-mediated disorder with still unclear pathogenesis. It is characterized by fibro-inflammatory tissue damage, IgG-4 positive plasma cells, and often elevated serum IgG4. This systemic disease can potentially affect every organ: pancreas, lymph nodes, lungs, meninges, vessels, kidneys (7). Renal involvement in IgG4 RKD can include tubulo-interstitial nephritis, membranous glomerulopathy (7–10% of cases), and obstructive disorders related to retroperitoneal fibrosis (17, 18). The epidemiology is still poorly described, but the disease appears more frequent in men over 50 years of age, with few cases of IgG4 RD having been reported in pediatric patients (19).

Therefore, the two patients herein described represent unique clinical features associated with pathogenetic *FOXP3* mutations that must be added to the list of clinical syndromes that may occur in this genetic contest. In a minority of cases, IPEX syndrome can indeed present an atypical phenotype and without the classic triad, but the incidence may be underestimated. Overall, our cases are consistent with the increasing evidence of atypical presentations of IPEX and also suggest that clinical manifestations are likely influenced by epigenetic factors or modifying genes (20). The genotype–phenotype correlation in IPEX is not clear: mutations in the DNA-binding site of *FOXP3* seem associated with poor outcomes (21), whereas there are mutations in the Fork-head domain and leucine-zipper domain (12) that are associated more frequently with mild phenotype or late onset. Case 2 is of particular interest in the context of epigenetic modifications since the asymptomatic brother of the proband and HSCT donor presented the same *FOXP3* mutations (unknown at the time of transplantation), thus supporting the evidence that clinical manifestations are unforeseeable, not related to the mutation type, and affected by some not yet identified regulatory mechanisms (22). Moreover, we performed Treg expression analysis on the blood cells of our patient after the hematopoietic stem cell *transplantation*, before and after the shift from tacrolimus to sirolimus, and on the donor in two different timings. The Treg phenotypes of the two brothers were different, and in our patient, the number of Treg was persistently very low, and Treg suppression activity was lower as compared to his sibling after therapeutic shift, strengthening our perspective (**Supplementary Materials 1 and 2**).

Data on kidney disease in IPEX syndrome are scarce: renal involvement is thought to occur in one-third of patients, sometimes as first manifestation of the disease (23). Interstitial nephritis, membranous glomerulopathy, and minimal change disease are the most common forms of renal injury (11).

The variety of kidney alteration in IPEX could be explained by the role of *FOXP3* on regulatory T cells. Tregs have, in fact, functional plasticity in response to different immune and genetic environment (24), and the dysregulation of Tregs could produce two major effects: one is the stimulation of IgG4 autoantibodies *versus* renal-specific antigens (PLA2R1 and THSD7A are the major) in membranous glomerulopathy, while the second potential effect of Tregs dysfunction is the increase of the release of cytokines by effector T cells that affect podocyte function (with the development of minimal change disease) (25). In IPEX syndrome, the pathogenesis of membranous glomerulopathy is consistent, with an imbalance between Treg and Th17 (26) that is due to a significant reduction of Tregs and *FOXP3* expression (27) in the presence of Th17 stable levels.

Parallel activation of the T helper 2 cells (Th2) lineage would promote IgG4 deposition (28). Th17/Treg imbalance may be implicated also in the pathogenesis of IgG4 RD and ALPS that are the two clinical settings herein described in association with FOXP3 mutations. In the former case (IgG4 RD), the increment of T helper 2 cells should stimulate IgG4-producing B cells (29) and upregulate Th17 (30, 31), resulting in a fibro-inflammatory disease involving various organs. Considering some recent evidence on the role of Treg in lymphoproliferative diseases, Mazarolles et al. investigated the Treg profiles in ALPS syndrome and found a reduced expression of CD3+CD4+CD25FOXP3+ Treg subsets (32). Further investigations are needed to support these initial evidence. A final point of interest is therapy. In the young boy with ALPS, immunosuppression was achieved with sirolimus, which spares normal Treg (33) and increases the suppressive function of IPEX patients’ Treg cells (34). Sirolimus has been extensively utilized for the control of IPEX syndrome (6), alone or in combination with steroids. This approach was functional to modify the outcome of ALPS and attenuate the clinical presentation of membranous glomerulopathy. In the second case, a steroid therapy was attempted without benefit at the start of symptoms; then, the child underwent HSCT that represents the gold standard for classic and severe forms of IPEX. Our patient had a good relief from the disease after HSCT, but he experienced a severe GvhD that impacted on residual kidney function, leading to the need for chronic kidney replacement therapy. Moreover, some complications experienced by our patients after HSCT (alopecia, candida infection, *etc.*) could be attributed to IPEX syndrome reactivation due to the donor HSCT’s FOXP3 mutations. Unfortunately, at the time of HSCT, a genetic diagnosis had not been reached, and in consideration of the urgency of the treatment, the selection of a HSCT donor was made based on available clinical data. With the improvement of massively parallel sequencing techniques and the reduction of the exam execution times, it will probably become mandatory to screen asymptomatic donors for the family variant prior to transplantation.

Overall, two cases with mutations of *FOXP3* have been described herein that had atypical clinical presentation in comparison to what it is expected in patients with this molecular feature, that is, IPEX syndrome. The renal picture of a primary autoimmune condition, membranous glomerulopathy in both cases, stimulated a genomic analysis that was central to recognize the atypical forms of IPEX. Epigenetic factors may have determined different clinical presentations in patients with classical *FOXP3* mutations, and the description of new phenotypes adds value to the genetic analysis that was central to the definition of the clinical settings. Common mechanisms of regulation of Treg/T helper cells function associated with FOXP3 function could explain the different clinical expressions of these cases that varied from IPEX to IgG4 RD and membranous glomerulopathy. Therapeutic strategies based on drugs modulating Tregs expression positively influenced the clinical outcome of the two patients herein described and should be considered in any other conditions associated with FOXP3 molecular defects.

## Data Availability Statement

The original contributions presented in the study are included in the article/**Supplementary Material**. Further inquiries can be directed to the corresponding author.

## Ethics Statement

The studies involving human participants were reviewed and approved by the Regional Ethic Committee of Liguria. Written informed consent to participate in this study was provided by the participants’ legal guardian/next of kin. Written informed consent was obtained from the individual(s)’ and minor(s)’ legal guardian/next of kin for the publication of any potentially identifiable images or data included in this article.

## Author Contributions

MG, ED, AA, LL, and EL had contributed to the conception of the study and wrote the paper. MM, MF, FP, PT, DF, and SG performed hematological and immunological investigations and reviewed the paper. AG, IC, and GC performed genetic analysis and interpretation of the data and reviewed the paper. DV and BC performed immunohistochemical and pathologic evaluation of biopsies. GG, EV, LN, and EF reviewed the manuscript and contributed to the final draft. All authors contributed to the article and approved the submitted version.

## Funding

The Institute Giannina Gaslini (trial sponsor) provided logistic and financial support to the trial through grants from the ministry of health (‘Cinque per mille of IRPEF-Finanziamentodellaricerca sanitaria’). The funder had no role in the design and conduct of the study; collection, management, analysis, and interpretation of the data; preparation, review, or approval of the manuscript; and decision to submit the manuscript for publication. We thank the “Associazione per la Cura del Bambino Nefropatico ONLUS” and the “Fondazione MalattieRenali del Bambino” ONLUS for supporting this study.

## Conflict of Interest

The authors declare that the research was conducted in the absence of any commercial or financial relationships that could be construed as a potential conflict of interest.

## Publisher’s Note

All claims expressed in this article are solely those of the authors and do not necessarily represent those of their affiliated organizations, or those of the publisher, the editors and the reviewers. Any product that may be evaluated in this article, or claim that may be made by its manufacturer, is not guaranteed or endorsed by the publisher.
