# Supplementary material for: Case Report: Atypical Manifestations Associated With FOXP3 Mutations. The “Fil Rouge” of Treg Between IPEX Features and Other Clinical Entities?
Source: Front Immunol. 2022 Apr 11;13:854749. doi: 10.3389/fimmu.2022.854749 (PMC9035826; doi:10.3389/fimmu.2022.854749)
Supplement: Supplementary file 1 [file Presentation_1.ppt]

## Slide 1
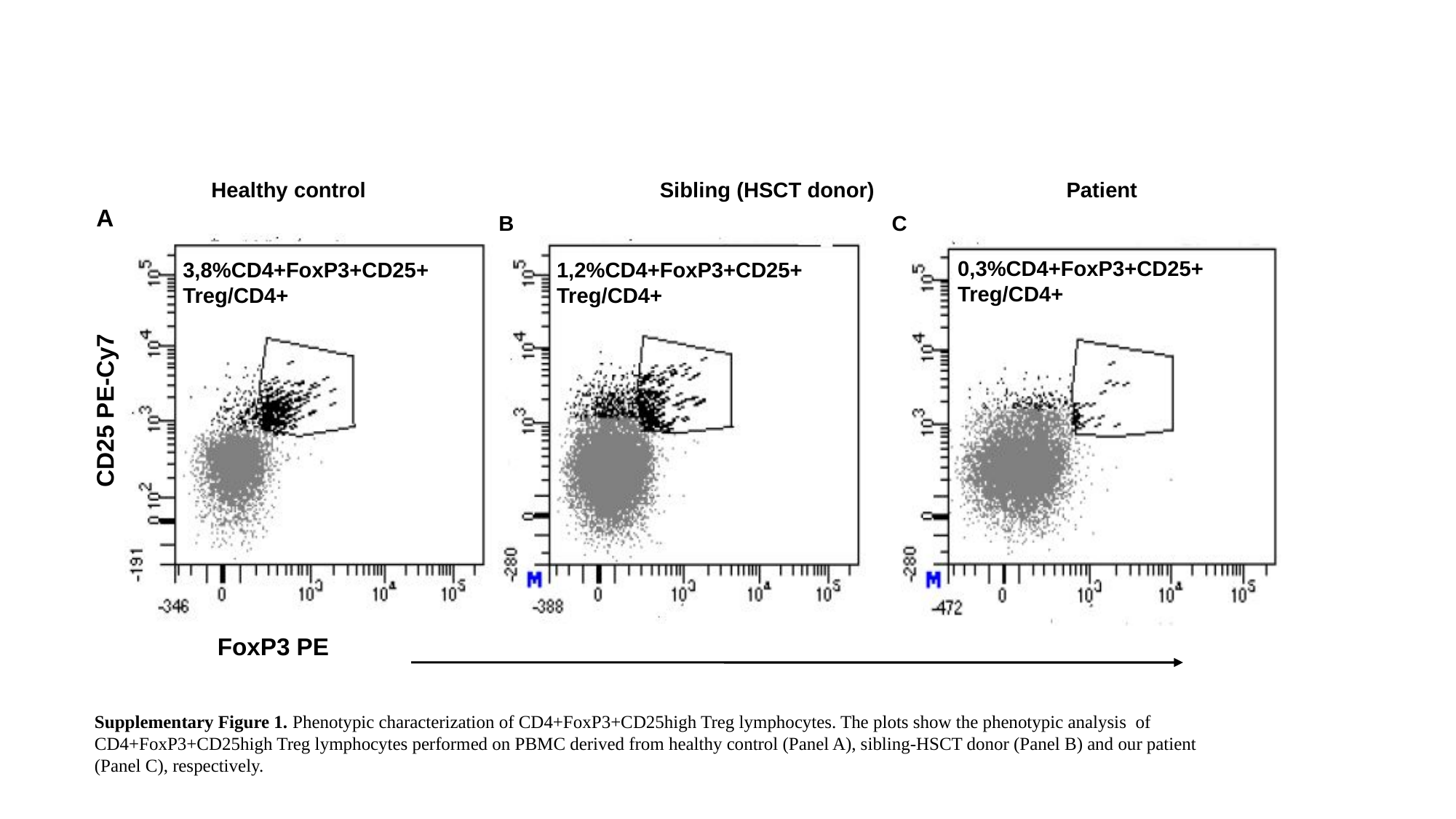

Healthy control 	 Sibling (HSCT donor) 		Patient
0,3%CD4+FoxP3+CD25+
Treg/CD4+
3,8%CD4+FoxP3+CD25+
Treg/CD4+
1,2%CD4+FoxP3+CD25+
Treg/CD4+
CD25 PE-Cy7
FoxP3 PE
A
B
C
Supplementary Figure 1. Phenotypic characterization of CD4+FoxP3+CD25high Treg lymphocytes. The plots show the phenotypic analysis of CD4+FoxP3+CD25high Treg lymphocytes performed on PBMC derived from healthy control (Panel A), sibling-HSCT donor (Panel B) and our patient (Panel C), respectively.
