# Supplementary material for: Case Report: Atypical Manifestations Associated With FOXP3 Mutations. The “Fil Rouge” of Treg Between IPEX Features and Other Clinical Entities?
Source: Front Immunol. 2022 Apr 11;13:854749. doi: 10.3389/fimmu.2022.854749 (PMC9035826; doi:10.3389/fimmu.2022.854749)
Supplement: Supplementary file 2 [file Presentation_2.ppt]

## Slide 1
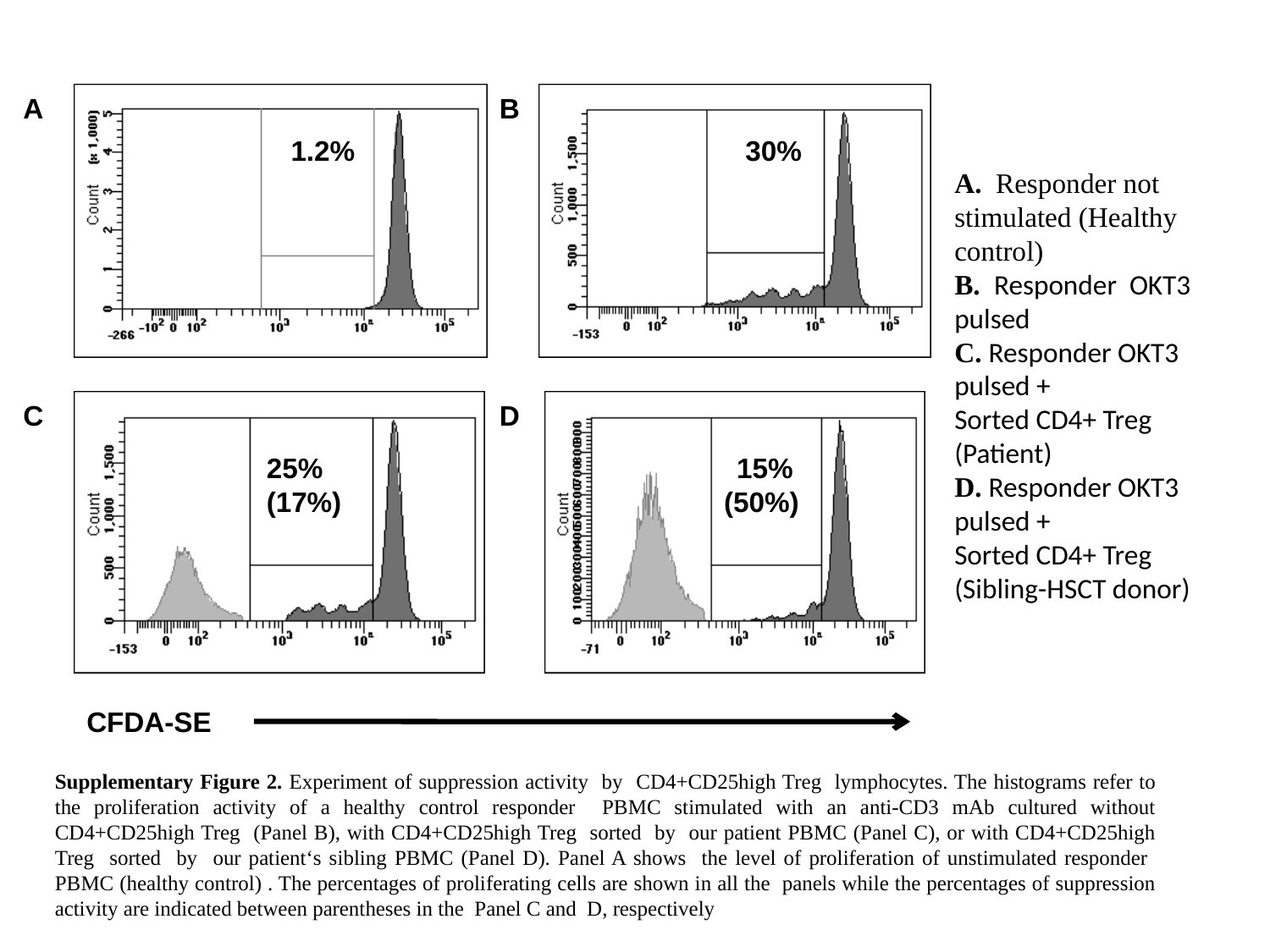

A
1.2% 30%
25% 15%
(17%) (50%)
CFDA-SE
B
C
D
A. Responder not stimulated (Healthy control)
B. Responder OKT3 pulsed
C. Responder OKT3 pulsed +
Sorted CD4+ Treg (Patient)
D. Responder OKT3 pulsed +
Sorted CD4+ Treg (Sibling-HSCT donor)
Supplementary Figure 2. Experiment of suppression activity by CD4+CD25high Treg lymphocytes. The histograms refer to the proliferation activity of a healthy control responder PBMC stimulated with an anti-CD3 mAb cultured without CD4+CD25high Treg (Panel B), with CD4+CD25high Treg sorted by our patient PBMC (Panel C), or with CD4+CD25high Treg sorted by our patient‘s sibling PBMC (Panel D). Panel A shows the level of proliferation of unstimulated responder PBMC (healthy control) . The percentages of proliferating cells are shown in all the panels while the percentages of suppression activity are indicated between parentheses in the Panel C and D, respectively
