# Supplementary material for: Case Report: Atypical Manifestations Associated With FOXP3 Mutations. The “Fil Rouge” of Treg Between IPEX Features and Other Clinical Entities?
Source: Front Immunol. 2022 Apr 11;13:854749. doi: 10.3389/fimmu.2022.854749 (PMC9035826; doi:10.3389/fimmu.2022.854749)
Supplement: Supplementary file 3 [file Table_1.doc]

Material and methods Supplementary Materials

*Monoclonal antibodies (mAbs) and immunofluorescence analyses*

Immunofluorescence analyses were performed on 1x10^6^ PBMC in 100 µl of PBS incubating with speciﬁc fluorochrome-conjugated mAbs. The following mAbs specific for surface markers were used: allophycocianin (APC)-H7-conjugated anti-CD45, APC-R700- conjugated anti-CD3, Peridinin Chlorophyll Protein Complex-cyanin 5.5 (PerCP-Cy5.5)-conjugated anti-CD4, phycoerythrin (PE)- cyanin (Cy) 7-conjugated anti-CD25 (BD Biosciences). To exclude dead cells, the samples were incubated with Aqua dead (Molecular Probes, Thermo Fisher) for 15 min at room temperature, before proceeding with surface staining. After surface staining, the cells were fixed and permeabilized by Transcription Buffer Set (BD Pharmingen) prior to perform intracellular staining for 30 min in the dark with PE- conjugated anti-FoxP3 (BD Biosciences). The cells were washed with 1 ml of PBS-BSA 0.01% and resuspended in 300 μl of PBS. The samples were analyzed by a BD Fortessa X20 flow cytometer (BD Biosciences) using the BD FACS Diva™ software version 8.0 (BD Biosciences).

*Purification of CD4+CD25high regulatory T lymphocytes*

Peripheral blood mononuclear cells (PBMC) were purified from heparinized blood samples from healthy control by centrifugation on Ficoll-Hypaque gradient (Biochrom AG, Berlin, Germany) for 30 min at 1,800 rpm.The CD4+CD25+ regulatory T cell isolation kit, human (Miltenyi Biotech, Bergisch Gladbach, Germany) was been used for purification of CD4+CD25+ regulatory T (Treg) cells. The purity of sorted cells was ≥ 95 % as demonstrated by flow cytometric analysis.

*Proliferation suppression assay*

The suppression activity was evaluated by monitoring the inhibition of dye dilution in PBMC from healthy donor stained before the test with carboxyfluorescein succinimidyl ester (CFDA-SE) (5 µM) (Molecular Probes, Invitrogen). After staining, the cells were pulsed with the anti-CD3 UCTH-1 mAb (5 µg/ml, BD Bioscience) and cultured for 5 days in a 96-well flat bottomed plate (1x 10^5^ cells/well) in the presence (or not) of the following CD4+CD25high Treg purified from the peripheral blood of patients. Then, the samples were washed in PBS and analyzed by a BD Fortessa X20 flow cytometer (BD Biosciences) using the BD FACS Diva™ software version 8.0 (BD Biosciences) in order to monitor the inhibition of dye dilution. Dead cells were excluded from analysis by adding 7-aminoactinomycin D (BD Biosciences) before the analysis. Suppression activity was expressed as percentage reduction of proliferation in the presence of CD4+CD25high Treg compared to the levels of proliferation observed in control cultures of PBMC (CD3 pulsed) alone. A suppression activity ≥25% was considered significant. This threshold was chosen based on the results achieved in a large historical cohort of more than 50 healthy subjects of both sexes with age ranging from 18 to 87 years. In healthy donors, CD8+ Treg suppression activity never fell below 25%.
